# Multiple Roles of LOXL2 in the Progression of Hepatocellular Carcinoma and Its Potential for Therapeutic Targeting

**DOI:** 10.3390/ijms241411745

**Published:** 2023-07-21

**Authors:** Jelena Radić, Bojana Kožik, Ivan Nikolić, Ivana Kolarov-Bjelobrk, Tijana Vasiljević, Bojana Vranjković, Sanja Despotović

**Affiliations:** 1 Faculty of Medicine, University of Novi Sad, 21137 Novi Sad, Serbia; jelena.radic@mf.uns.ac.rs (J.R.); ivan.nikolic@mf.uns.ac.rs (I.N.); ivana.kolarov-bjelobrk@mf.uns.ac.rs (I.K.-B.); tijana.vasiljevic@mf.uns.ac.rs (T.V.); 2 Department of Medical Oncology, Oncology Institute of Vojvodina, 21204 Sremska Kamenica, Serbia; bojana.vranjkovic@onk.ns.ac.rs; 3 Laboratory for Radiobiology and Molecular Genetics, Vinča Institute of Nuclear Sciences—National Institute of the Republic of Serbia, University of Belgrade, 11100 Belgrade, Serbia; bojana86@vin.bg.ac.rs; 4 Department of Pathology, Oncology Institute of Vojvodina, 21204 Sremska Kamenica, Serbia; 5 Institute of Histology and Embryology, Faculty of Medicine, University of Belgrade, 11000 Belgrade, Serbia

**Keywords:** hepatocellular carcinoma (HCC), LOXL2, tumor microenvironment (TME), extracellular matrix (ECM), LOXL2 inhibitors, target therapy

## Abstract

LOXL2, a copper-dependent amine oxidase, has emerged as a promising therapeutic target in hepatocellular carcinoma (HCC). Increased LOXL2 expression in HCC has been linked with an aggressive phenotype and represents a poor prognostic factor. Here, we focus on the mechanisms through which LOXL2 orchestrates multiple oncogenic functions in HCC development. We performed a review of the current knowledge on the roles LOXL2 performs in the modulation of the HCC tumor microenvironment, formation of premetastatic niches, and epithelial–mesenchymal transition. We also highlighted the complex interplay between LOXL2 and hypoxia, angiogenesis, and vasculogenic mimicry in HCC. At the end of the review, we summarize the current LOXL2 inhibitors and discuss their potential in HCC precision treatment.

## 1. Introduction

Hepatocellular carcinoma (HCC) is the most common type of primary liver cancer, accounting for more than 80% of cases [1]. With incidence and mortality rates increasing worldwide, it represents a global healthcare concern [1,2]. The underlying process in the development of HCC is chronic liver damage leading to cirrhosis, which is most often caused by chronic viral hepatitis (hepatitis B and C), alcohol abuse, and metabolic dysfunction-associated steatotic liver disease (MASLD) [3]. Around 80% of HCC arises from a cirrhotic liver, and among patients with cirrhosis of any etiology, one-third will develop HCC [1,4,5]. The pathogenesis of HCC is a complex multistep process that usually starts with liver injury and inflammation. Chronic liver inflammation triggers a fibrous process, which over time can progress to cirrhosis and is characterized by the extensive disruption of liver tissue architecture. Prolonged inflammation and cirrhosis create precancerous settings resulting in the generation of dysplastic foci and dysplastic nodules, which further accumulate genetic/epigenetic alterations, resulting in the development of hepatocellular carcinoma [6].

The most appropriate therapeutic option is determined based on the TNM tumor stage, degree of background liver damage, and the patient’s overall health [7]. The recommended therapy for an early-stage HCC is surgical resection, liver transplantation, or radiofrequency ablation. Transarterial chemoembolization (TACE) and radiation therapy, alone or in combination with systemic therapy, are used for intermediate-stage HCCs, while systemic therapy (chemotherapy, molecular targeted therapy, immunotherapy, and gene therapy) is the treatment of choice for advanced-stage HCCs [7,8]. Unfortunately, due to the asymptomatic nature of early-stage HCC, more than 60% of cases are detected in the intermediate or advanced stage, where curative therapeutic options are limited [9]. The backbone of systemic therapy for HCC is sorafenib and other multi-kinase inhibitors [7,10,11,12]. Moreover, ramucirumab, a vascular endothelial growth factor (VEGF) inhibitor and immune checkpoint inhibitors (nivolumab and pembrolizumab) are being increasingly used, either separately or in combination [7,13]. Although the choices for therapeutic strategies in advanced HCC are rapidly expanding, they provide a variable but still limited extension of survival, cause a wide range of side effects, and ultimately, lead to the development of tumor resistance, which is recognized as one of the biggest problems in the treatment of HCC [10]. 

The multitude of underlying mechanisms that are responsible for the development of resistance to therapy by HCC is not completely understood, although it has been shown that tumor microenvironment (TME) plays an important role. The tumor microenvironment has a crucial role in hepatocarcinogenesis and directly participates in the regulation of liver fibrosis and tumor-progressive processes, such as epithelial–mesenchymal transition (EMT), extracellular matrix (ECM) remodeling, migration, invasion, and metastasis [14]. Unraveling the complex interactions within the tumor microenvironment and targeting the components of the TME, such as ECM remodeling enzymes, might serve as a valuable strategy to improve the current therapeutic options and develop novel ones while attempting to re-sensitize resistant tumors to existing therapeutic agents [10,15,16].

In the last fifteen years, lysyl oxidase-like 2 (LOXL2) has emerged as one of the major mediators between tumor cells and TME. This research has implicated the involvement of LOXL2 in every step of tumor progression [17,18,19,20,21,22]. The involvement of LOXL2 has been reported in the regulation of cancer cell proliferation, epithelial–mesenchymal transition, migration, extravasation, and creating premetastatic niches at distant sites, as reviewed by Zhang et al. [23], Lin et al. [24], and Wen et al. [25]. The mechanisms through which LOXL2 affects tumor invasiveness can be used as a typical model of solid cancer progression and spreading [26]. In addition, LOXL2 regulates tumor-induced angiogenesis [27,28,29] and mediates the interaction between cancer cells and cancer-associated fibroblasts (CAF), and macrophages [22,30,31]. Taken together, LOXL2 represents a multifunctional protein, which is enrolled in almost every step of solid tumor propagation. In this review, we will summarize the multiple roles of LOXL2 in the progression of HCC and its potential for therapeutic targeting.

## 2. LOXL2 Introduction: LOX Family, Structure, and LOXL2 Function

**Figure 1:**
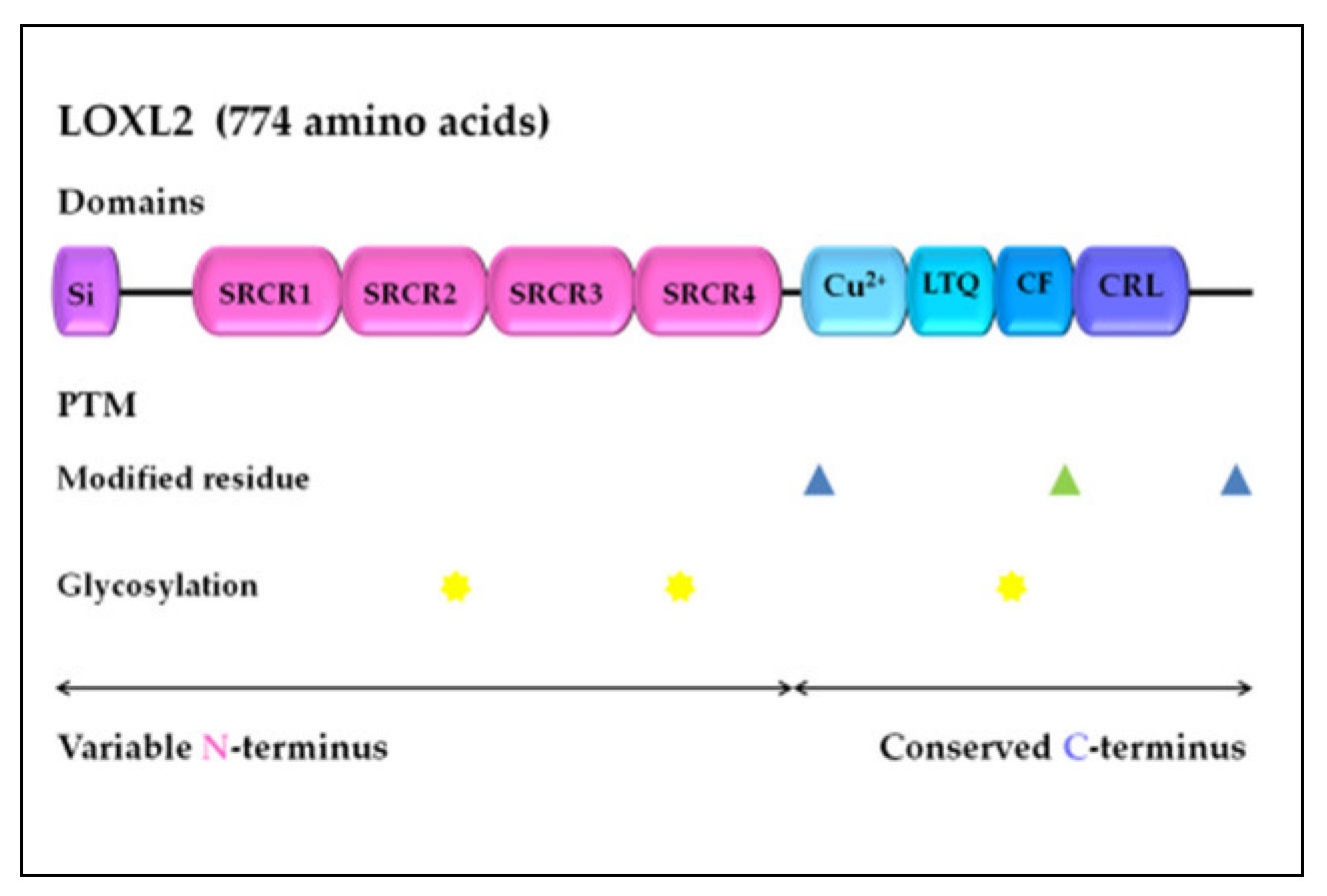
LOXL2 protein structure. Arrows indicate the variable N-terminal domain and conserved C-terminal domain consists of several different functional domains, represented by boxes. Post-translational modifications of LOXL2 were obtained using UniProt. Modified residues are represented by blue triangles (phosphoserine at 601 and 722) and green triangle (2′,4′,5′-topaquinone at 689), while N-linked glycans at N288, N455, and N644 are marked with yellow stars. Si, signal peptide; SRCR, scavenger receptor cysteine-rich domain; Cu^2+^, copper binding domain; LTQ, lysine tyrosylquinone cofactor residues; CF, cofactor formation; CRL, cytokine receptor-like domain; PTM, post-translational modifications. The figure drawing is not-to-scale (for detailed LOXL2 structure, see Meier et al. [44]).

LOXL2 is a secreted and copper-dependent amine oxidase that belongs to the lysyl oxidase (LOX) family, which consists of five homologous members: LOX and LOX-like l–4 (LOXL1–4) proteins [32,33,34,35,36,37]. The primary function of the LOX family enzymes is to catalyze the cross-linking of elastin and collagen by oxidation, which is essential for maintaining the rigidity, stability, and remodeling of the extracellular matrix (ECM) [38,39]. The human *LOXL2* gene is positioned on chromosome 8p21-22 and encodes a 774 amino acid protein [26,40]. Structurally, the LOXL2 protein contains a variable N-terminal region and a highly conserved C-terminal region with catalytic activity (Figure 1). The catalytic domains at the C-terminus are conserved among LOX family members and consist of the copper-binding domain, lysyl tyrosine quinone (LTQ) element, which is required for cofactor formation, and a cytokine receptor-like (CRL) domain [41,42]. The LOXL2 N-terminal domain is more variable and includes a signal peptide and four scavenger receptor cysteine-rich (SRCR) elements, which are responsible for protein–protein interactions [41,43].

**Figure 2:**
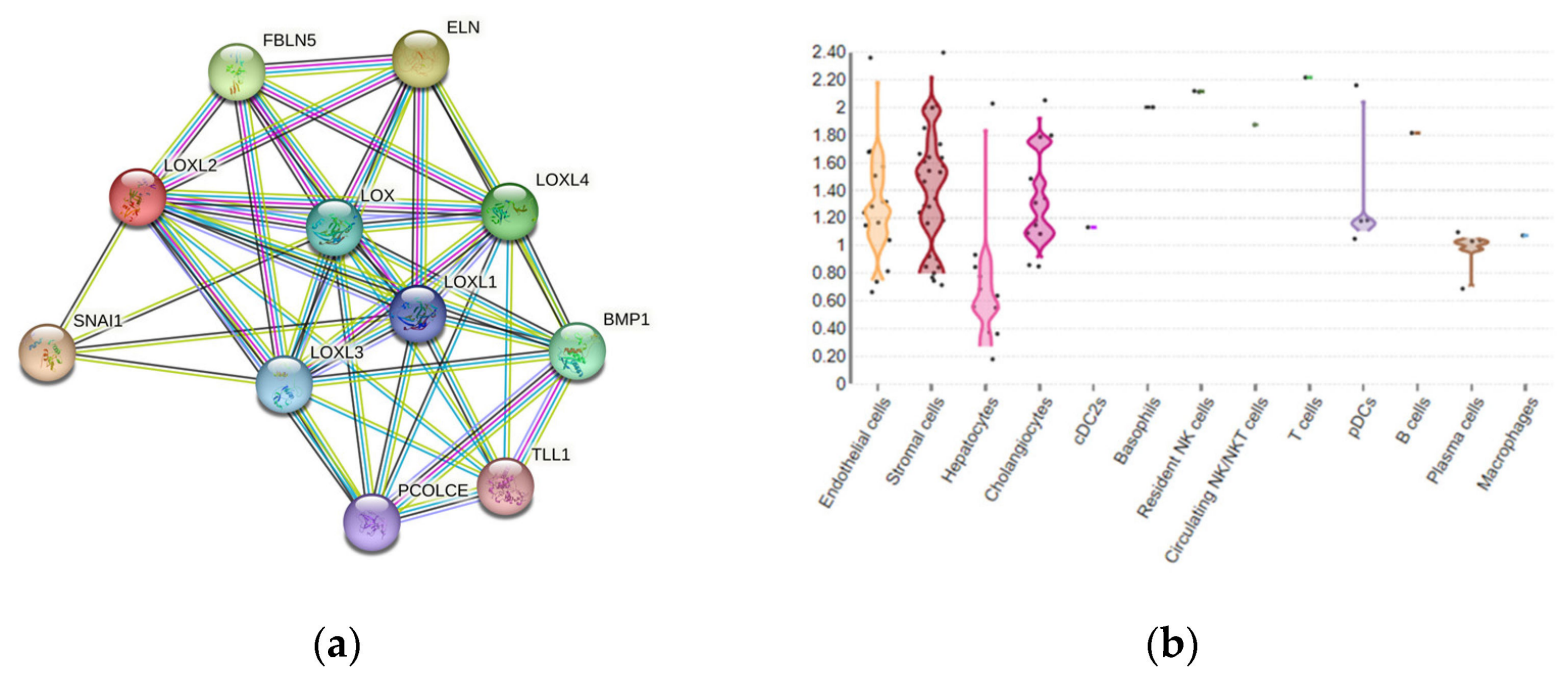
(**a**) LOXL2 protein interactome. Available on the String platform [52]. SNAI1, zinc finger protein involved in the induction of the epithelial–mesenchymal transition (EMT); ELN, elastin; FBLN5, fibulin-5, which is essential for elastic fiber formation; PCOLCE, procollagen C-endopeptidase enhancer 1, which binds to the C-terminal propeptide of type I procollagen; TLL1, Tolloid-like protein 1, protease that processes procollagen C-propeptides; BMP1, bone morphogenetic protein 1, which cleaves the C-terminal propeptides of procollagen I, II, and III. (**b**) LOXL2 spatial expression in healthy liver cells. Available from the Liver Cell Atlas online dataset, Reprinted/adapted with permission from Ref. [51]. 2023, Charlotte Scott and Martin Guilliams [51].

The existence of several different protein domains in the LOX family of enzymes implies their multiple biological functions [26], in addition to maintaining the structure of the ECM. The LOX and LOXL1–4 proteins are enrolled in other biological processes, including embryogenesis and development [42,45,46]. Although LOXL2 is a secretory protein, it is also distributed in the intracellular compartments and within the nucleus, thereby can exert its intracellular and extracellular activities through various downstream pathways (Figure 2a) [25]. LOXL2 participates in the structural maintenance of the ECM in fibrotic tissues, such as in the liver tissue [47,48,49]. LOXL2-specific tissue expression among various liver cell compartments can be estimated using multi-omics datasets, as presented in Guilliams et al. [50], which detects the overall expression of LOXL2 in 0.7% of healthy adult liver cells, dominantly in stromal and endothelial cells, as well as in hepatocytes and cholangiocytes (Figure 2b) [51].

Altered expression and activation of LOX family members can affect the tissue microenvironment, which is implicated in many pathological conditions, including tissue fibrosis, atherosclerosis, and tumor development [53]. Previous studies have indicated that LOXL2 expression is regulated at the transcriptional and post-transcriptional levels [54,55,56], as well as by alternative splicing [57,58], and through an interaction with micro-RNA molecules [59,60,61]. LOXL2 protein activity can also be regulated by post-translational modifications, specifically glycosylation, while an aberrant level of glycosylation has been linked to human malignancies [62]. N-glycosylation is typical in membrane and secretory proteins, while the recombinant human LOXL2 protein, from human embryonic kidney cells, is glycosylated at N288, N455, and N644 (N-linked glycans), which significantly improves its stability and secretion (Figure 1) [63].

In recent years, an increasing number of studies have revealed the upregulation of LOXL2 expression in various solid tumors [64]. However, the mechanism of LOXL2-mediated tumor progression has mostly been investigated in breast cancer [64,65]. Interestingly, in breast cancer cell lines overexpressing LOXL2, two forms of LOXL2 were generated: a non-glycosylated intracellular form (~75-kDa) and an extracellular form (~100-kDa), which had been glycosylated at N455 and N644 [66]. Moreover, it has been shown that breast carcinomas have a more prominent invasive ability, via the actions of intracellular LOXL2, compared to the extracellular effects of LOXL2 in ECM remodeling, thereby suggesting that LOXL2 has specific intra- and extracellular functions [64,65,66]. Tumor progression induced by enhanced LOXL2 expression has been well documented in various digestive system-related cancers, including HCC [23]; thus, its extra- and intracellular functions should be further explored as a potential novel therapeutic target for HCC.

## 3. LOXL2 Expression in HCC and Correlation with Clinical Parameters

LOXL2 is overexpressed in human HCC tissue compared to healthy liver tissue, at both the mRNA and protein levels [17,24,67,68,69,70,71,72]. LOXL2 protein expression has been shown to correlate to the amount of fibrosis in the tumor stroma and was more pronounced in the cytoplasm of cancer cells directly adjacent to a fibrous stroma, compared to centrally located cancer cells [71]. LOXL2 expression was positively correlated with the direct invasion of adjacent liver tissue [17], increased frequent portal vein invasion, poor tumor differentiation, and more advanced TNM stage [71]. Moreover, LOXL2 expression in HCC patients, especially high-risk HCCs (HCC tumor size > 5 cm, HCCs with portal vein invasion, poor differentiation, and TNM stage II or III), correlated with shorter overall survival, disease-free survival, disease-specific survival, and extrahepatic recurrence-free survival [24,69,70,71]. Hypoxia, chronic inflammation, and fibrosis have all been shown to induce LOXL2 expression in HCC [15,17]. Additionally, in human HCC tissue samples, the co-expression of LOXL2 and carbon anhydrase IX, which is a hypoxia-related biomarker, showed a better correlation with prognostic parameters compared to LOXL2 alone [71]. Interestingly, LOXL2 levels were significantly higher in the serum of HCC patients than in the serum of non-HCC patients [17], making LOXL2 a promising biomarker for HCC.

## 4. LOXL2 in the Regulation of Tumor Microenvironment and Formation of Premetastatic Niches

TME in HCC is a complex and dynamic landscape that is composed of tumor cells and various host elements, including cells and extracellular ECM components, which surround and interact with tumor cells, as well as with each other in multiple ways. These interactions dramatically alter every step of HCC progression, and its phenotype, and modulate the treatment response [14]. Numerous host cellular components are involved in shaping the TME of HCC, including activated hepatic stellate cells (HSCs), cancer-associated fibroblasts (CAFs), cells of innate and acquired immunity, sinusoidal endothelial cells, and bone marrow-derived cells (BMDCs) [73,74]. These cells interact with the ECM, an abundant meshwork of proteins, carbohydrates, and water, which is far from the passive network, yet a very dynamic environment involved in every step of tumor development and progression [74,75,76].

The ECM constituents can be broadly defined as fibers (collagen, elastic, and reticular) and ground substances (proteoglycans, glycoproteins, and glycosaminoglycans). In the healthy liver, the ECM represents a small compartment situated within the liver capsule, portal tracts, around the central vein, and in the space of Disse. The ECM serves as an anchor to the hepatocytes and stromal cells and influences their polarity, differentiation, shape, and migration, thereby representing a reservoir of signaling molecules, and playing a role in intercellular communication [77]. Due to an imbalance between excessive production and decreased degradation of certain components, the ECM undergoes profound remodeling during HCC development and progression [16,77].

**Scheme 1:**
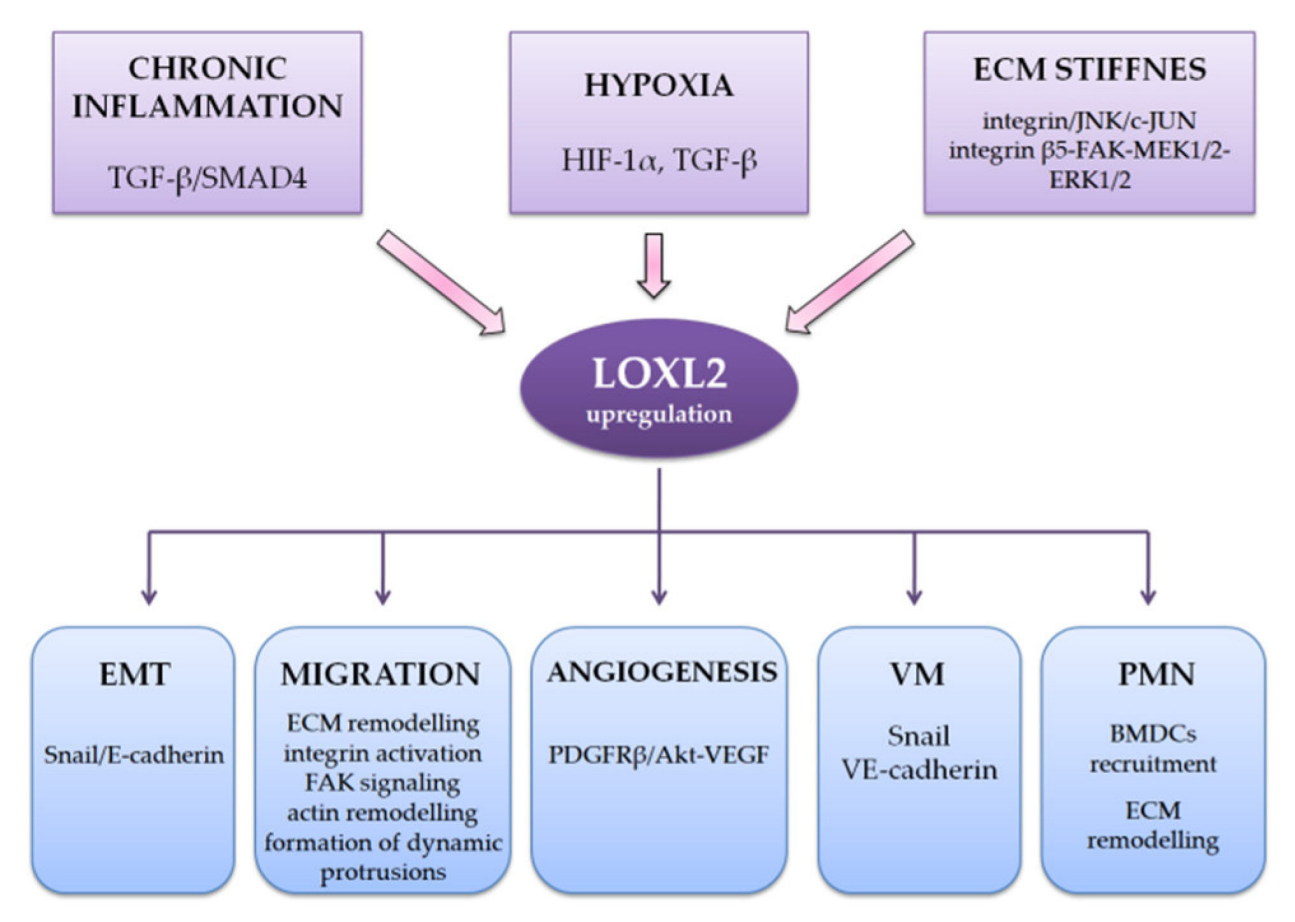
Interaction with HCC tumor microenvironment and roles in HCC progression. ECM, extracellular matrix; EMT, epithelial–mesenchymal transition; VM, vasculogenic mimicry; PMN, premetastatic niches.

The most important factors determining the specificity of the HCC TME are inflammation, hypoxia with vascular remodeling, and fibrosis [15,73,74]. HCC is a prototype of an inflammation-associated carcinoma, where by around 90% of HCCs arise in the settings of chronic inflammation caused by HBV and HCV, or MASLD [73,78]. Hypoxia, described in more detail later in this review, has been traditionally reported as one of the typical features of HCC [79]. As previously mentioned, more than 80% of HCC arises in the settings of pronounced fibrosis or cirrhosis, which develop as a hepatic response to chronic liver injury and significantly influence the TME [1,4,5]. The most abundant ECM component in the fibrotic or cirrhotic liver is collagen type I, which is mainly synthesized by activated HSCs and CAFs, although other ECM components are becoming increasingly recognized in this process. Thus, numerous studies involving HCC cell lines and animal models have shown that chronic inflammation (induced by activating TGF-β/SMAD4 pathway), hypoxia (dominantly via HIF induction but also through the induction of TGF-β), and stiff fibrous ECM (via integrin/JNK/c-JUN pathway) in the HCC microenvironment can upregulate LOXL2 levels (Scheme 1) [17,80]. 

Secreted LOXL2 catalyzes covalent cross-linking of collagen, which results in the formation of thick collagen fibers [17]. The increased deposition of collagen fibers and LOXL2-mediated collagen cross-linking results in a further increase in ECM stiffness, which changes the biochemical and biomechanical ECM properties and, as it has been shown in cell culture experiments, facilitates cancer cell migration and metastasis, epithelial–mesenchymal transition, stemness, and proliferation of HCC cells [15,81,82,83], which all correlate to a poor prognosis [84]. Additionally, it has been demonstrated that a LOXL2-mediated increase in ECM stiffness impairs the efficacy of chemotherapeutics 5FU and sorafenib through a cell adhesion-mediated drug resistance mechanism and by limiting drug delivery [15,83].

Periostin, a matricellular glycoprotein secreted mainly by activated HSCs, has emerged as a marker of poor prognosis and increased metastatic potential in HCC [85,86,87,88]. Periostin plays an important role in liver fibrosis and ECM stiffness by stimulating the cross-linking of collagen I and influencing both the density and diameter of the collagen fibers. Periostin binds to BMP-1 and enhances its incorporation into the fibronectin matrix, which facilitates pro-LOX cleavage and its activation, which leads to collagen cross-linking [89]. In vitro studies and experiments based on mouse models have shown that periostin significantly induces LOXL2 expression in HSCs, at the mRNA and protein levels [90]. Adding to the complexity, periostin treatment induced the secretion of collagen type I, fibronectin, and TGF-β in cultured HSCs. Periostin performs its function via integrin αvβ3 and PI3K signaling, which leads to TGF-β-independent SMAD2/3 phosphorylation and the induction of LOXL2 transcription. 

In addition to regulating fibrosis, periostin also induces epithelial–mesenchymal transition in hepatocytes [90], a process whereby LOXL2 plays an important role, which is described further below. In addition to interacting with collagen type I and periostin, LOXL2 also interacts with many other partners from matrisome; however, the significance of these interactions and their role in HCC progression has yet to be investigated [91].

The migration of cancer cells, which underlies the local invasion and represents the initial event in the metastatic cascade, is a complex multistep process. It involves integrin-mediated mechano-signal transduction from components of the ECM to the actin cytoskeleton, which results in cytoskeletal remodeling and the formation of lamellipodia or invadosomes at the leading edge of cancer cells [77,92,93]. Sensing biomechanical ECM properties includes the formation of focal adhesion complexes, which consist of clusters of integrins, signaling proteins (such as talin, paxillin, and tensin), the activation of several kinases, and ultimately, modified gene transcription [77]. It has been shown that LOXL2-mediated collagen cross-linking enhanced focal adhesion signaling and the formation of prominent and dynamic cell protrusions [14,16,94] in HCC cell lines. LOXL2 influences actin remodeling, which is a prerequisite for the formation of dynamic protrusions and cell migration, by inducing integrin activation and the activity of crucial components of the focal adhesions, including FAK, Rho kinase ROCK, paxillin, and vinculin (Scheme 1) [15,17]. Animal experiments in vivo showed that LOXL2 enhanced infiltrative tumor growth, and thus, stimulated local invasion and the formation of intrahepatic and distant metastases [17].

### 4.1. LOXL2 and Cancer-Associated Fibroblasts (CAFs)

One of the crucial components of the HCC tumor microenvironment is cancer-associated fibroblasts (CAFs), which morphologically and phenotypically resemble myofibroblasts [95]. CAFs are most commonly derived from the hepatic stellate cells (HSCs) and to a lesser extent from the resident tissue fibroblasts, mesenchymal stem cells, or endothelial cells [96]. The relationship between CAFs and tumor cells has already been extensively studied. CAFs promote HCC development, growth, angiogenesis, local invasion, and metastasis through the secretion of numerous growth factors and cytokines, by interacting with other cellular elements in the HCC tumor microenvironment, and through ECM remodeling [16,96,97]. On the other hand, tumor cells secrete cytokines, such as TGF-β and PDGF, which stimulates the transdifferentiation and activation of CAFs [16,97]. In chronic liver diseases, which precede HCC development, the chronic damage of hepatocytes, its subsequent regenerative response, and various inflammatory cytokines, all transform quiescent HSCs and fibroblasts into activated myofibroblasts. Myofibroblasts synthesize numerous ECM components, including collagen type I and growth factors, which leads to parenchyme reorganization, fibrosis, and vascular remodeling [98,99,100]. Cell culture experiments have shown that CAFs upregulate LOXL2 levels in HCC cells, thus, promoting their invasion and metastatic capacity [101]. In addition, studies using human HCC samples and cell culture experiments have shown that the CAF-derived chemokine CCL5 can activate the HIF-1α/ZEB1 cascade and promote metastatic spread [102]. As previously mentioned, HIF-1α is a potent inducer of LOXL2 expression [17]. On the other hand, some cell culture experiments have demonstrated that HCC cells upregulate LOXL2 expression in CAFs by secreting various soluble mediators, thus, creating a positive feedback loop in the HCC TME, which facilitates tumor invasion and metastatic spread [101].

### 4.2. LOXL2 and Tumor-Associated Macrophages (TAMs)

Adding further to the complexity of the HCC tumor microenvironment, cell culture experiments showed that increased ECM stiffness induced the polarization of macrophages towards the M2 phenotype and increased their LOXL2 expression. In the same study, authors showed that the integrin β5–FAK–MEK1/2–ERK1/2 pathway mediated ECM stiffness and induced HIF-1α upregulation, while HIF-1α further stimulated LOXL2 expression in M2 polarized macrophages [31,67]. M2 macrophages phenotypically resemble tumor-associated macrophages (most tumor-associated macrophages show M2 polarization). They are also involved in the progression of HCC via the secretion of growth- and angiogenic factors, ECM remodeling, and by inducing an immunosuppressive state [103,104]. A study by Klepfish et al. supported these findings. This study showed that LOXL2 inhibition in fibrotic liver disease led to the accumulation of reparative monocyte-derived macrophages, which secrete matrix metalloproteinases (MMPs) and degrade fibrous collagen, thereby paving the way to reverse liver fibrosis and many of its deleterious effects [105].

### 4.3. LOXL2 in the Formation of Premetastatic Niches (PNM)

Today it is known that before the metastatic spread has occurred, tumors actively induce changes at distant sites, which leads to the formation of a permissive microenvironment and will facilitate the survival and proliferation of circulating tumor cells upon arrival. These modified microenvironments are named premetastatic niches (PNM) [106]. The two most commonly described changes in the premetastatic niches are the recruitment of bone marrow-derived cells (BMDC) and ECM remodeling. Cell culture experiments showed that LOXL2 was secreted from HCC cells, which were stimulated by stiff TME, and this further influenced the seeding, motility, and invasion of the BMDCs. Moreover, secreted LOXL2-induced MMP9 and fibronectin expression in lung fibroblasts, which suggests it plays a role in ECM remodeling (Scheme 1) [80]. The same research indicated AKT pathway involvement in the LOXL2-induced upregulation of fibronectin in lung fibroblasts [80]. Secreted LOXL2-induced chemokine CXCL-12 (also called stromal cell-derived factor-1) expression in lung fibroblasts, there by promoting the recruitment of endothelial progenitor cells, chemotaxis of inflammatory and circulating cancer cells, and their proliferation and survival [107]. The number of HCC cells that adhered to the LOXL2-exposed lung fibroblasts was significantly higher than in those without LOXL2 exposure, further indicating the role of LOXL2 in the seeding of circulating cancer cells in the premetastatic niches [80]. Experiments conducted in vivo have revealed that mice bearing HCC tumors with LOXL2 inhibition demonstrate a significantly reduced frequency of lung metastases [17,108]. Furthermore, mice with HCC tumors with LOXL2 inhibition showed a significant reduction in collagen cross-linking and BMDCs infiltration in the lungs, all of which indicates the important role of LOXL2 in the formation of the premetastatic niches [17,108,109].

## 5. LOXL2 Role in Epithelial–Mesenchymal Transition

Another important event in the metastatic spread of carcinomas is the process of the epithelial–mesenchymal transition, whereby the cell, either partially or completely, acquires a mesenchymal phenotype, which allows for cell migration. This further facilitates the detachment of cancer cells from the tissue of origin and its invasion through surrounding tissues, intravasation and extravasation, and dissemination to distant sites [110,111]. Previous studies have demonstrated the relationship between LOXL2 intracellular activities and the induction of the epithelial–mesenchymal transition in different tumor types [112,113,114]. The epithelial–mesenchymal transition usually starts with the downregulation of E-cadherin expression [115]. It is important to note that the Snail transcription factor is one of the direct E-cadherin repressors, and its elevated expression has been detected in many invasive carcinomas [116]. The mechanism responsible for LOXL2-induced epithelial–mesenchymal transition includes the inhibition of E-cadherin expression via interaction with the Snail transcription factor (Scheme 1) [116]. At first, the study by Peinado et al. [116] revealed that the amine oxidase activity of LOXL2 regulates Snail stability, and subsequently inhibits the expression of E-cadherin. However, further analysis showed that LOXL2 point mutants with inactivated catalytic activity also induced epithelial–mesenchymal transition by physically interacting with Snail1 [117]. Park et al. [108] revealed that the FoxM1b transcription factor, which plays an important role in HCC progression, increases LOXL2 expression through directly binding to the promoter of the *LOXL2* gene, which consequently activates the AKT–Snail1 pathway and triggers epithelial–mesenchymal transition in mouse-derived HCC cell lines. This process boosts liver fibrosis and HCC metastasis in vivo. In addition, a previous study on human HCC samples confirmed a significant correlation between LOXL2 expression and the epithelial-mesenchymal transition status measured by the vimentin to E-cadherin ratio [118].

## 6. LOXL2 and Hypoxia, Angiogenesis, and Vasculogenic Mimicry

Hypoxia is a frequent characteristic of the tumor microenvironment in solid tumors, including HCC [79]. Moreover, standard palliative HCC treatments impair afferent blood flow and suppress cancer growth, simultaneously creating a hypoxic microenvironment [17]. Hypoxic conditions trigger activation of the hypoxia-inducible factor, HIF-1α, which acts as a transcription activator of numerous genes, including LOXL2, by binding to the hypoxia-responsive element (HRE) within the LOXL2 promoter (Scheme 1) [119,120]. In experiments using human HCC cell lines, Wong et al. demonstrated that HIF-1α enhanced LOXL2 expression both directly and indirectly via the TGF–β/SMAD4 pathway [17]. Moreover, Tse et al. [121] studied the interaction between the HBV viral oncoprotein HBx with its transactivator activity and HIF-1α in mice HCC lines, and the results revealed that HBx overexpression induces HIF-1α stabilization, which further activates LOXL2 expression and HCC metastatic spread in transgenic HBx mice. Results published by Fan et al. [122] showed that LOXL2 also acts as an activator of HIF-1α expression in HCC cell lines, through the Snail–FBP1 pathway. Collectively, these findings indicate the significant role of LOXL2 in HCC progression induced by hypoxia.

HCC represents one of the most vascularized solid tumors, which is one of the main features of highly malignant tumors [123,124]. Hypoxic conditions in the TME induce de novo vascularization or angiogenesis, which additionally enhances tumor growth, invasion, and metastatic spread (Scheme 1) [125]. VEGF is the promoter of classical tumor angiogenesis and its expression can be regulated by LOXL2 via the PDGFRβ/Akt axis [126]. It has been shown that extracellular LOXL2 inhibition in tumor xenografts significantly reduces VEGF-A and CXCL12 signaling [127]. CXCL12 acts synergistically with VEGF in promoting the migration and proliferation of endothelial cells [128]. The standard management of advanced HCC using first-line antiangiogenic agents (sorafenib, lenvatinib, and regorafenib) represents a promising treatment approach, although the patient’s response can be unsatisfactory [129,130,131]. Antiangiogenic therapies further cause oxygen and nutrient deprivation within HCC tissue and may induce an alternative method of tumor neovascularization called vasculogenic mimicry [132]. Vasculogenic mimicry is characterized by the formation of vessels lined by tumor cells and supported by ECM elements [133]. In HCC, vasculogenic mimicry is associated with tumor aggressiveness and poor clinical outcome [134]. Recent studies provide evidence that LOXL2 is also involved in vasculogenic mimicry during the progression of HCC [29,67]. Wang et al. [67] investigated the mechanisms underlying the enhanced levels of LOXL2 in a hypoxic TME of HCC cells and noted that the activation of LOXL2 by HIF-1α also promoted vasculogenic mimicry, which helped HCC progression. Further in vitro and in vivo analysis of vasculogenic mimicry in HCC revealed that LOXL2 induces vasculogenic mimicry and the metastatic spread of the tumor by upregulating Snail1 and VE-cadherin [29]. A better understanding of LOXL2-mediated vasculogenic mimicry may enable the development of a new treatment strategy, which could compensate for the side effects of classical antiangiogenic drugs used in HCC treatment. In the study by Li et al., from 2022, the authors demonstrated that the knockdown of LOXL2 effectively inhibited the expression of the anti-apoptosis proteins BIRC3 and MDM2 and induced cell apoptosis and cell cycle arrest in HCC cell lines [135]. However, further research is needed to fully understand the complexities of the LOXL2-mediated apoptosis pathways and their implications in HCC [29,67,123,124,125,126,127,128,129,130,131,132,133,134,135].

## 7. LOXL2 and micro-RNAs in HCC

Recent studies have revealed that micro-RNAs (miRs) may be differentially expressed in HCC and that some of them function as negative epigenetic regulators of LOXL2, thereby affecting the LOXL2 TME-mediated processes. Wong et al. demonstrated that miR-26 and miR-29 suppress LOXL2 in HCC by directly binding to the 3′UTR of LOXL2 mRNA [17]. Specifically, miR29-a has been identified as a negative regulator of hypoxia-responsive genes, such as *HIF-1α*, *VEGFa*, *LOX*, and *LOXL2* in HCC cell lines and HCC tissue functional studies [69,136]. Repression of miR-29a has been mediated by MYC; MYC amplification represents one of the earliest events in HCC development [137,138]. Recent bioinformatics analysis proposed hsa-miR-192-5p as a potential LOXL2 regulator in HCC; however, more in vivo studies are required to clarify these interactions between LOXL2 and specific micro-RNAs in the pathogenesis of HCC [139].

## 8. LOXL2 as Potential Target for Treatment of HCC

The well-established treatment options for patients with HCC have limited clinical efficacy in the late stages of HCC due to severe fibrosis and cirrhosis [140]. Therefore, chemoresistant HCC cases require novel combined treatment strategies. Since LOXL2 is unquestionably involved in almost every step of HCC progression and dissemination, this protein fulfills conditions that are required for a potential target in developing molecular anticancer therapy. Considering the LOXL2 protein structure, two approaches for targeting this protein were considered in drug development studies. The first was based on the fact that LOXL2 demands copper ions for enzyme activity, and the second was to target the lysine tyrosylquinone region (LTQ), which possesses a cofactor binding function [141]. Designing highly selective LOXL2 inhibitors is promising and could be beneficial for HCC patients with LOXL2 overexpression since the downregulation of LOXL2 expression reduces tumor cell invasiveness and metastatic spread [142].

The first monoclonal LOXL2 antibody, AB0023, which specifically binds to the fourth SRCR domain was developed in 2010 and has been effective in clinical studies (Table 1) [49,127,143]. Its efficacy was demonstrated in both tumor xenografts as well as in liver fibrosis models [49,127]. On the other hand, simtuzumab, a humanized LOXL2 antibody (AB0024), has not achieved satisfactory results in clinical trials for fibrotic diseases and several solid tumors [144,145,146]. The lack of clinical effectiveness can be explained by the fact that specific antibody inhibitors probably only deactivate extracellular LOXL2, while the intracellular functions of LOXL2 remain preserved due to incomplete antibody internalization or the compensatory activity of other LOX family members [25,82]. In the search for more selective LOXL2 inhibitors, most efforts have been toward designing a small molecule inhibitor in order to increase specificity and efficacy, and to minimize its side effects [141]. According to the data available on the PHAROS web interface for exploring target/ligand interactions [147], a query for ”LOXL2” resulted in 227 active ligands (ChEMBL compounds with an activity cutoff of <30 nM), although clinical trials that focus on testing LOXL2 based-drugs for HCC are still lacking.

One of the most explored LOX inhibitors is β-aminopropionitrile (BAPN), a small irreversible inhibitor that blocks the catalytic activity of all LOX members and displays specific affinity to LOXL2 [141,148]. Previous studies have shown significant tumor suppressive effects by BAPN in several solid tumors and tumor-cell lines [149,150,151,152,153]. Moreover, some studies demonstrated that BAPN affects the TME and impedes the interaction between cancer-associated fibroblasts and gastric cancer cells, resulting in a reduction in the frequency and size of the liver metastasis [154]. In HCC, BAPN inhibited tumor growth and angiogenesis in vivo and hampered the migration and invasion of HCC cell lines [70,118]. According to the study by Liu et al. [155], BAPN also showed an ameliorative effect in the CCl4-induced model of liver fibrosis. The first selective LOXL2 inhibitor, LOXL2-IN-1 hydrochloride, demonstrated potential for use in HCC treatment since it suppressed Snail1, HIF-1α, and VEGF, the main drivers of HCC progression, as mentioned above [24]. In recent years, the second generation of small-molecular-weight haloallylamine-based LOXL2 inhibitors was explored, including PXS-5338, PXS-5382, and PXS-5878, which showed promising results in inhibiting the catalytic activity of LOXL2 [156]. A dual LOXL2/LOXL3 inhibitor, PXS-5153A, was designed in 2019 by Schilter et al. [157] and also demonstrated ameliorating effects and a significant improvement in liver fibrosis.

**Table 1:** LOXL2 inhibitors for HCC and liver fibrosis.

Type	Agent	Target	References
monoclonal antibody	AB0023	LOXL2	[46,121,122]
	AB0024	LOXL2	[123,124,125]
small-molecule inhibitor	BAPN	LOX/LOXL1-4	[100,133,134]
	LOXL2-IN-1	LOXL2	[122]
	PXS-5338	LOXL2	[156]
	PXS-5382	LOXL2	[156]
	PXS-5878	LOXL2	[156]
	PXS-5153A	LOXL2/LOXL3	[157]
	(2-chloropyridin-4-yl) methenamine	LOXL2	[158]

BAPN, beta-aminopropionitrile; PXS, (2S)-propane-1,2-diyl dihexadecanoate.

Currently, the most promising results on the potential utility of LOXL2 inhibitors in HCC treatment have been published by Gong et al. [15]. The authors demonstrated that a highly selective LOXL2 inhibitor, namely (2-chloropyridin-4-yl) methenamine [158], in combination with 5FU and sorafenib treatment, significantly decreased the tumor size in a mouse model study. Moreover, HCC cells treated with this agent showed a significantly better response to 5FU and sorafenib treatment [15]. These results suggest that LOXL2 inhibition has the potential to be more successful in patients with HCC in late fibrotic stages with the concomitant application of 5FU and sorafenib. However, these observations in animal studies need to be validated in further clinical studies.

## 9. Conclusions

Although treatment strategies for HCC are continuously developing, the treatment of intermediate- and advanced-stage HCC remains a major clinical challenge. This indicates the need for novel molecular therapeutic targets. Data summarized in this review emphasize the multiple roles of LOXL2 in crucial processes involved in HCC progression: remodeling of the HCC TME, stimulating the migration of cancer cells, and therefore, local invasion, the formation of premetastatic niches and metastasis, epithelial–mesenchymal transition, angiogenesis, and vasculogenic mimicry. All these features make LOXL2 an attractive potential target for innovative therapy design. Further research on clinical–pharmacological solutions of LOXL2-based therapy is warranted. More comprehensive analyses are needed to establish the role of other LOX family members and mutual interactions among the LOX members in the HCC pathogenesis. Targeting LOXL2 must be approached from the perspective of the matrisome, as a whole. In other words, detailed knowledge of multiple LOXL2 interactions with various tumor microenvironment components in HCC and other malignancies, as well as in healthy tissues, is necessary to understand the many effects resulting from LOXL2 inhibition. In conclusion, the development of a highly selective LOXL2 inhibitor compatible with the concomitant application of standard HCC chemotherapy might represent a future direction for a personalized treatment strategy for HCC. 

## Data Availability

No new data were created or analyzed in this study. Data sharing is not applicable to this article.

## Abbreviations

HCC	Hepatocellular carcinoma;
LOXL2	Lysyloxidase-like 2;
HBV	Hepatitis B virus;
HCV	Hepatitis C virus;
MASLD	Metabolic dysfunction-associated steatotic liver disease;
TACE	Transarterialchemoembolization;
VEGF	Vascular endothelial growth factor;
TME	Tumor microenvironment;
CAF	Cancer-associated fibroblasts;
ECM	Extracellular matrix;
LTQ	Lysine tyrosylquinone;
ELN	Elastin;
FBLN5	Fibulin-5;
PCOLCE	Procollagen C-endopeptidase enhancer 1;
TLL1	Tolloid-like protein 1;
BMP	Bone morphogenic protein 1;
CAIX	Carbon anhydrase IX;
HSC	Hepatic stellate cells;
BMDC	Bone marrow-derived cells;
TGF-ß	Transforming growth factor-beta;
HIF-1α	Hypoxia-inducible factor 1 alpha;
JNK-c	Jun N-terminal kinase;
5FU	5-fluorouracil;
FAK	Focaladhesion kinase;
ROCK	Rho-associated protein kinase;
CCL5	Chemokine ligand 5;
ZEB1	Zinc finger E-box-binding homeobox 1;
MEK1/2	Mitogen-activated protein kinase 1/2;
ERK 1/2	Extracellular signal-regulated kinase 1/2;
MMP	Matrix metalloproteinase
HRE	Hypoxia responsive element;
FBP1	Fructose-1,6-biphosphatase protein 1;
PDGFRß	Platelet-derived growth factor receptor beta;
miR	micro-RNA;
BAPN	ß-aminopropionitrile.
